# Supplementary material for: Facilitating Mitophagy via Pink1/Parkin2 Signaling Is Essential for the Neuroprotective Effect of β-Caryophyllene against CIR-Induced Neuronal Injury
Source: Brain Sci. 2022 Jun 30;12(7):868. doi: 10.3390/brainsci12070868 (PMC9313355; doi:10.3390/brainsci12070868)
Supplement: Supplementary file 1 [file brainsci-12-00868-s001.zip › brainsci-1779005-supplementary.pdf]

Table S1: Summary of 36 recognized mitophagy -associated genes.

Gene	Type
ULK1	Mitophagy
FUNDC1	Mitophagy
PGAM5	Mitophagy
TOMM40	Mitophagy
TOMM5	Mitophagy
TOMM20	Mitophagy
TOMM7	Mitophagy
TOMM22	Mitophagy
MFN2	Mitophagy
PINK1	Mitophagy
SQSTM1	Mitophagy
VDAC1	Mitophagy
VDAC2	Mitophagy
VDAC3	Mitophagy
ATG12	Mitophagy
ATG5	Mitophagy
CISD2	Mitophagy
CISD1	Mitophagy
COX4I1	Mitophagy
CISD3	Mitophagy
SOD1	Mitophagy
BECN1	Mitophagy
ITPR1	Mitophagy
WFS1	Mitophagy
SFXN4	Mitophagy
CIAPIN1	Mitophagy
MRFAP1	Mitophagy
HTRA2	Mitophagy
MFN1	Mitophagy
PARK2	Mitophagy
PARK7	Mitophagy
GPR37	Mitophagy
HSP90AA1	Mitophagy
SNCA	Mitophagy
UBE2L3	Mitophagy
SNCAIP	Mitophagy
